# Two Independent Mutations in *ADAMTS17* Are Associated with Primary Open Angle Glaucoma in the Basset Hound and Basset Fauve de Bretagne Breeds of Dog

**DOI:** 10.1371/journal.pone.0140436

**Published:** 2015-10-16

**Authors:** James A. C. Oliver, Oliver P. Forman, Louise Pettitt, Cathryn S. Mellersh

**Affiliations:** Department of Canine Genetics Research, Centre for Preventive Medicine, Animal Health Trust, Newmarket, Suffolk, United Kingdom; Casey Eye Institute, UNITED STATES

## Abstract

**Purpose:**

Mutations in *ADAMTS10* (CFA20) have previously been associated with primary open angle glaucoma (POAG) in the Beagle and Norwegian Elkhound. The closely related gene, *ADAMTS17*, has also been associated with several different ocular phenotypes in multiple breeds of dog, including primary lens luxation and POAG. We investigated *ADAMTS17* as a candidate gene for POAG in the Basset Hound and Basset Fauve de Bretagne dog breeds.

**Methods:**

We performed *ADAMTS17* exon resequencing in three Basset Hounds and three Basset Fauve de Bretagne dogs with POAG. Identified variants were genotyped in additional sample cohorts of both breeds and dogs of other breeds to confirm their association with disease.

**Results:**

All affected Basset Hounds were homozygous for a 19 bp deletion in exon 2 that alters the reading frame and is predicted to lead to a truncated protein. Fifty clinically unaffected Basset Hounds were genotyped for this mutation and all were either heterozygous or homozygous for the wild type allele. Genotyping of 223 Basset Hounds recruited for a different study revealed a mutation frequency of 0.081 and predicted frequency of affected dogs in the population to be 0.007. Based on the entire genotyping dataset the association statistic for the POAG-associated deletion was p = 1.26 x 10^−10^. All affected Basset Fauve de Bretagne dogs were homozygous for a missense mutation in exon 11 causing a glycine to serine amino acid substitution (G519S) in the disintegrin-like domain of *ADAMTS17* which is predicted to alter protein function. Unaffected Basset Fauve de Bretagne dogs were either heterozygous for the mutation (5/24) or homozygous for the wild type allele (19/24). Based on the entire genotyping dataset the association statistic for the POAG-associated deletion was p = 2.80 x 10^−7^. Genotyping of 85 dogs of unrelated breeds and 90 dogs of related breeds for this variant was negative.

**Conclusion:**

This report documents strong associations between two independent *ADAMTS17* mutations and POAG in two different dog breeds.

## Introduction

Glaucoma is a heterogeneous disease which is usually associated with defects within the trabecular meshwork (TM) and anterior chamber which lead to obstruction of aqueous humour outflow, elevation of intraocular pressure (IOP) and progressive retinal ganglion cell death and optic nerve degeneration. In humans, glaucoma is the most common cause of irreversible blindness worldwide [1]. Glaucoma is described as primary if it occurs in the absence of any antecedent ocular disease and secondary if it occurs as a result of a concurrent observable ocular condition. Primary glaucoma can be further classified into primary open angle glaucoma (POAG) and primary closed angle glaucoma (PCAG) on the basis of the appearance of the iridocorneal angle. POAG is the more common form of primary glaucoma in man, will affect 58.9 million people by 2020 and is typically characterised by adult onset and chronic IOP-dependent progression [1,2].

In man, a number of studies have indicated the familial nature of POAG and support the role of genetic factors in its pathogenesis [3–6]. POAG in man is generally considered to be a complex genetic disorder and a minority of POAG cases can be explained by mutations in single genes. That said, multiple families are known to be affected by Mendelian forms of POAG and linkage analyses have identified multiple loci which segregate with disease [7–9], leading to the identification of causal mutations within several genes including myocillin (*MYOC*), optineurin (*OPTN*) and WD repeat domain 36 (*WDR36*) [10–12].

In dogs, POAG is a relatively rare condition although its high prevalence in specific breeds suggests an inherited aetiology. Affected breeds include the Beagle and Norwegian Elkhound [13–17]. In both these breeds POAG is an autosomal recessive condition caused by two separate mutations in *ADAMTS10* [14–16]. Further research in the Beagle has shown that the *ADAMTS10* mutation responsible for POAG is also associated with an inherently weaker and biochemically distinct posterior sclera before clinical evidence of optic nerve damage becomes apparent [18]. Mutations in a closely related gene, *ADAMTS17*, are responsible for primary lens luxation in multiple dog breeds and POAG in the Petit Basset Griffon Vendeen [19–21]. In man, homozygous mutations in *ADAMTSL4*, *ADAMTS10*, *ADAMTS17* cause various ocular phenotypes including ectopia lentis, myopia and glaucoma [22–24]. Furthermore, ADAMTS4 has been shown to be important in aqueous humour outflow in human and porcine eyes [25]. These studies suggest that the ADAMTS and ADAMTSL gene families play an important role in ocular function and demonstrate how mutations within the same gene can cause multiple ocular phenotypes. In this manuscript, we report our investigation of *ADAMTS17* as a candidate gene for POAG in two additional dog breeds—the Basset Hound and Basset Fauve de Bretagne.

## Materials and Methods

### Sample Collection and Nucleic Acid Extraction

All experiments were conducted in accordance with the ARVO statement for the Use of Animals in Ophthalmic and Vision Research and approved by the Animal Health Trust’s Research and Ethical Approval Committee. All dogs were pets and ophthalmological examination and buccal mucosal swabbing were only performed after informed and written owner consent.

226 Basset Hounds and 27 Basset Fauve de Bretagne dogs underwent ophthalmic examination by a board-certified veterinary ophthalmologist which included slit-lamp biomicroscopy, direct and indirect ophthalmoscopy, rebound tonometry and gonioscopy. These examinations are non-painful and non-invasive. Gonioscopy was performed only following application of topical anaesthetic to ensure no breach of animal welfare. All Basset Hounds were recruited for examination for a separate study and were believed by the owners to be free from clinical ophthalmic disease at the time of examination. These dogs were examined at different events and locations across the United Kingdom to ensure as representative a cross-section of the breed population was sampled as possible. No attempt was made to target Basset Hounds of any particular lineage; all dogs that were volunteered by their owners were examined and included in the study, regardless of their sex, age or pedigree. Affected Basset Fauve de Bretagne dogs presented as clinical cases whereas the unaffected dogs of this breed were recruited to screen for evidence of POAG.

POAG was diagnosed in 3 Basset Hound (2 males, 1 female) and 3 Basset Fauve de Bretagne dogs (1 male, 2 females). All affected dogs had different owners and resided in different locations and, at the time of sampling, were not known to be generally related. The mean age (±SD) of the POAG affected dogs was 49.33±5.44 months (Basset Hounds) and 68.33±12.66 months (Basset Fauve de Bretagnes). Inclusion criteria for dogs with POAG were; absence of an identifiable cause of secondary glaucoma, open iridocorneal angles on gonioscopy, elevated IOP (>25mmHg), buphthalmos and lens subluxation. 223 Basset Hound and 24 Basset Fauve de Bretagne dogs were found to be free from all clinical signs of POAG. The 223 clinically unaffected Basset Hounds had a mean age of 51.96±2.61 months and 85 (38%) were male and 138 (62%) were female. The 24 clinically unaffected Basset Fauve de Bretagnes had a mean age of 124.38±78.1 months and 12 (50%) were male and 12 (50%) were female. DNA was extracted from buccal mucosal swabs from each dog using the QIAmp^®^ DNA blood Midi Kit (Qiagen, Manchester, UK) according to manufacturer’s instructions.

### *ADAMTS10* Candidate Variant Genotyping

All POAG-affected Basset Hounds and Basset Fauve de Bretagne dogs were genotyped for the two previously published canine POAG-associated variants using primers and thermal cycling parameters as previously described [14,16]. Sanger sequencing methodology, was used with Bigdye v3.1 chemistry (Life Technologies Ltd, Paisley, UK). Sequencing products were separated on an ABI3130xl genetic analyser and data analysed using the Staden Gap4 software package (http://staden.sourceforge.net/).

### *ADAMTS17* Resequencing

Primer pairs were designed to amplify the coding sequence and flanking splices sites for all 23 canine *ADAMTS17* exons using gene sequences derived from the Ensembl genome browser (http://www.ensembl.org/index.html) (S1 Table). Resequencing of the exons was performed after PCR amplification of genomic DNA in all POAG cases. PCRs were carried out in 12 μl reactions consisting of 0.6 U Qiagen HotStarTaq DNA polymerase and 1 x PCR buffer (Qiagen, Manchester, UK), 200 μM dNTPs (Fisher Scientific—UK Ltd, Loughborough, UK), 0.83 μM forward and reverse primer (Integrated DNA Technologies, Leuven, Beligum) and 10 ng template genomic DNA. The Qiagen Q solution additive (1 x) was used for GC rich amplicons (exons 1 & 2) (Qiagen, Manchester, UK). Reaction mixtures were subjected to a thermal cycling program of 95°C for 10 min, followed by 35 cycles of 95°C for 30 s, 30 s at the annealing temperature (S1 Table) and 72°C for 1 min and a final elongation stage of 72°C for 10 min. *ADAMTS17* exome libraries for Illumina sequencing were made using the Nextera XT DNA Library Preparation Kit as per the manufacturer’s instructions (Illumina, San Diego, USA). Sequencing of all POAG cases was performed on the Illumina MiSeq platform, generating a dataset of 75 bp paired-end reads. Next generation sequencing results were confirmed for all POAG cases by Sanger sequencing.

### Sequencing Data Analysis

Sequence reads were aligned to the canine reference genome assembly (CanFam3.1) using the Burrows-Wheeler Alignment tool (BWA) [26]. Read alignments were visualised using the Integrated Genome Viewer and the data manually browsed for variants [27]. The potential pathogenicity of non-synonymous variants was tested with the SIFT bioinformatics tool [28]. Sanger sequencing results were analysed using the Staden Gap4 software package (http://staden.sourceforge.net/).

### Genotyping of the *ADAMTS17* Candidate Causal Variants

Candidate causal variants were genotyped using either a fragment length analysis or Sanger sequencing approach. Primer pairs were designed to amplify the candidate causal variants using gene sequences derived from the UCSC bioinformatics site (https://genome.ucsc.edu/) (S2 Table). For the Basset Hound, 12 μl PCR reactions consisted of 0.5 U Qiagen HotStarTaq DNA polymerase, 1 x Qiagen PCR buffer and 1 x Qiagen Q solution (Qiagen, Manchester, UK), 200 μM dNTPs (Fisher Scientific—UK Ltd, Loughborough, UK), 0.4 μM forward and reverse primer (Integrated DNA Technologies, Leuven, Belgium) and 10 ng template genomic DNA. Reaction mixtures were subjected to a thermal cycling program of 95°C for 5 min, followed by 35 cycles of 95°C for 30 s, 30 s at an annealing temperature of 60°C (Table 1) and 72°C for 30 s and a final elongation stage of 72°C for 30 min. For the Basset Fauve de Bretagne dogs the 12 μL reactions consisted of 0.25 U Qiagen HotStarTaq DNA polymerase and 1 x Qiagen PCR buffer (Qiagen, Manchester, UK), 200 μM dNTPs (Fisher Scientific—UK Ltd, Loughborough, UK), 0.83 μM forward and reverse primer (Integrated DNA Technologies, Leuven, Belgium) and 10 ng template genomic DNA. Reaction mixtures were subjected to a thermal cycling program of 95°C for 5 min, followed by 35 cycles of 95°C for 30 s, 57°C for 30 s and 72°C for 30 s and a final elongation stage of 72°C for 5 min. Basset Hounds were genotyped for the 19 bp deletion using capillary electrophoresis of 5’ 6FAM labelled PCR products on ABI3130xl genetic analysers, and data scored using GeneMapper v4.0 (Life Technologies Ltd, Paisley, UK). Basset Fauve de Bretagne dogs were genotyped for the candidate SNP using a standard Sanger sequencing methodology, using Bigdye v3.1 chemistry (Life Technologies Ltd, Paisley, UK). Sequencing products were separated on an ABI3130xl genetic analyser and data analysed using the Staden Gap4 software package (http://staden.sourceforge.net/). Additional genotyping and verification of the Sanger sequencing results was performed using a TaqMan allelic discrimination approach (Life Technologies, Paisley, UK) [29]. Primer details are provided in S3 Table. Reactions were carried out in 8 μl volumes consisting of 4 μl Kapa probe fast (Kapa Biosystems), 0.2 μl 40x probe mix, 2 μl genomic DNA and 1.8 μl ultrapure water. Cycling parameters were 40 cycles of 95°C for 3 s and 60°C for 15 s. Primer and probe sequences are listed in S3 Table. Mutant allele frequencies were calculated and the frequency of affected dogs calculated assuming the alleles were in Hardy-Weinberg equilibrium within the population. Based on the entire genotyping data set for each breed, POAG-associated association statistics for candidate variants were calculated using PLINK (http://pngu.mgh.harvard.edu/~purcell/plink/anal.shtml).

**Table 1:** Published mutations and their phenotypes in canine and human *ADAMTS17*.

Species (breed or ethnicity)	Ocular Phenotype	Systemic Phenotype	Exon/intron	Mutation	Nature of mutation	State of zygosity	Reference
Man (Saudi Arabian)	Lenticular myopia, ectopia lentis, spherophakia, glaucoma	Short stature	Exon 18	c.2458_2459insG	Frameshift	Homozygous	
Man (Saudi Arabian)	Lenticular myopia, ectopia lentis, spherophakia	Short stature, brachydactyly, joint stiffness	Intron 12	c.1721+1G>A	Splice site	Homozygous	[22]
Man (Saudi Arabian)	Lenticular myopia, ectopia lentis, spherophakia,	Short stature	Exon 4	c.760C>T	Nonsense	Homozygous	[22]
Man (Saudi Arabian)	Spherophakia	Short stature	Exon 4	c.652delG	Frameshift	Homozygous	[41]
Man (Tunisan)	Microspherophakia, myopia	Short stature, congenital ichthyosis	Exon 1–3	106.96 Kb deletion	Deletion	Homozygous	[42]
Man (Indian)	Microspherophakia	Short stature, brachydactyly	Intron 5	c.873+1G>T	Splice site	Homozygous	[23]
Dog (multiple breeds)	Primary lens luxation	None	Intron 10	GT>AT	Splice site	Homozygous	[19]
Dog (Petit Basset Griffon Vendeen)	POAG	None	Intron 12	Large scale genetic rearrangement	Inversion	Homozygous	[21]
Dog (Basset Hound)	POAG	None	Exon 2	19 bp deletion	Deletion	Homozygous	Present study
Dog (Basset Fauve de Bretagne)	POAG	None	Exon 11	c.1552G>A	Missense	Homozygous	Present study

## Results

### *ADAMTS10* genotyping

All POAG-affected Basset Hounds and Basset Fauve de Bretagne dogs were homozygous for the wild type alleles at the chromosomal locations of both previously published POAG-associated variants.

### *ADAMTS17* Resequencing

#### Basset Hound

**Fig 1:**
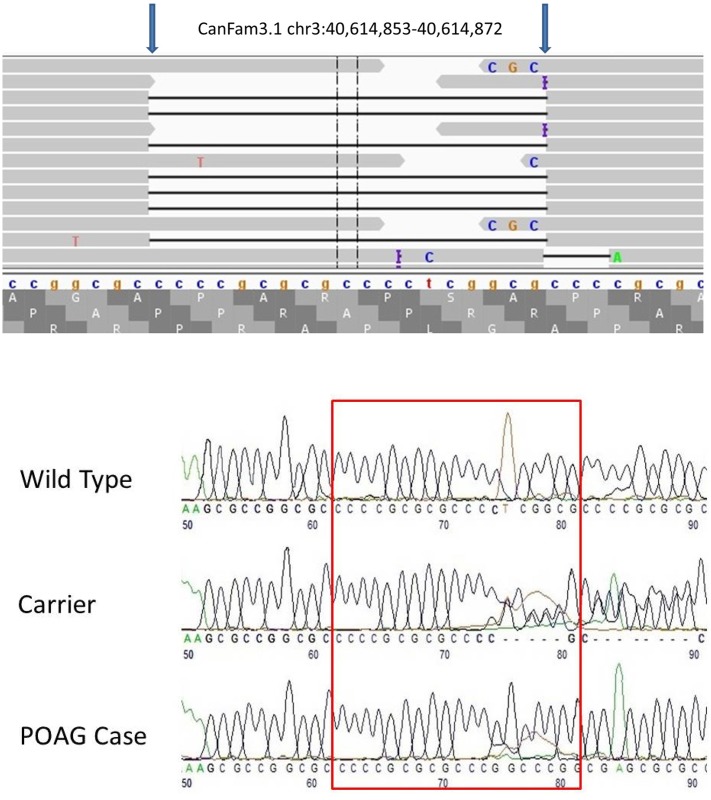
Next generation and Sanger sequencing results depicting the site of 19bp deletion in the Basset Hound with POAG. **a)** IGV view of the 19 bp deletion in a Basset Hound affected with POAG. The location of this homozygous deletion is chr3:40,614,853–40,614,872 (CanFam3.1). b) Electropherograms depicting the site of the deletion. The red box delineates the affected sequence in wild type, carrier and POAG-affected dogs.

**Fig 2:**
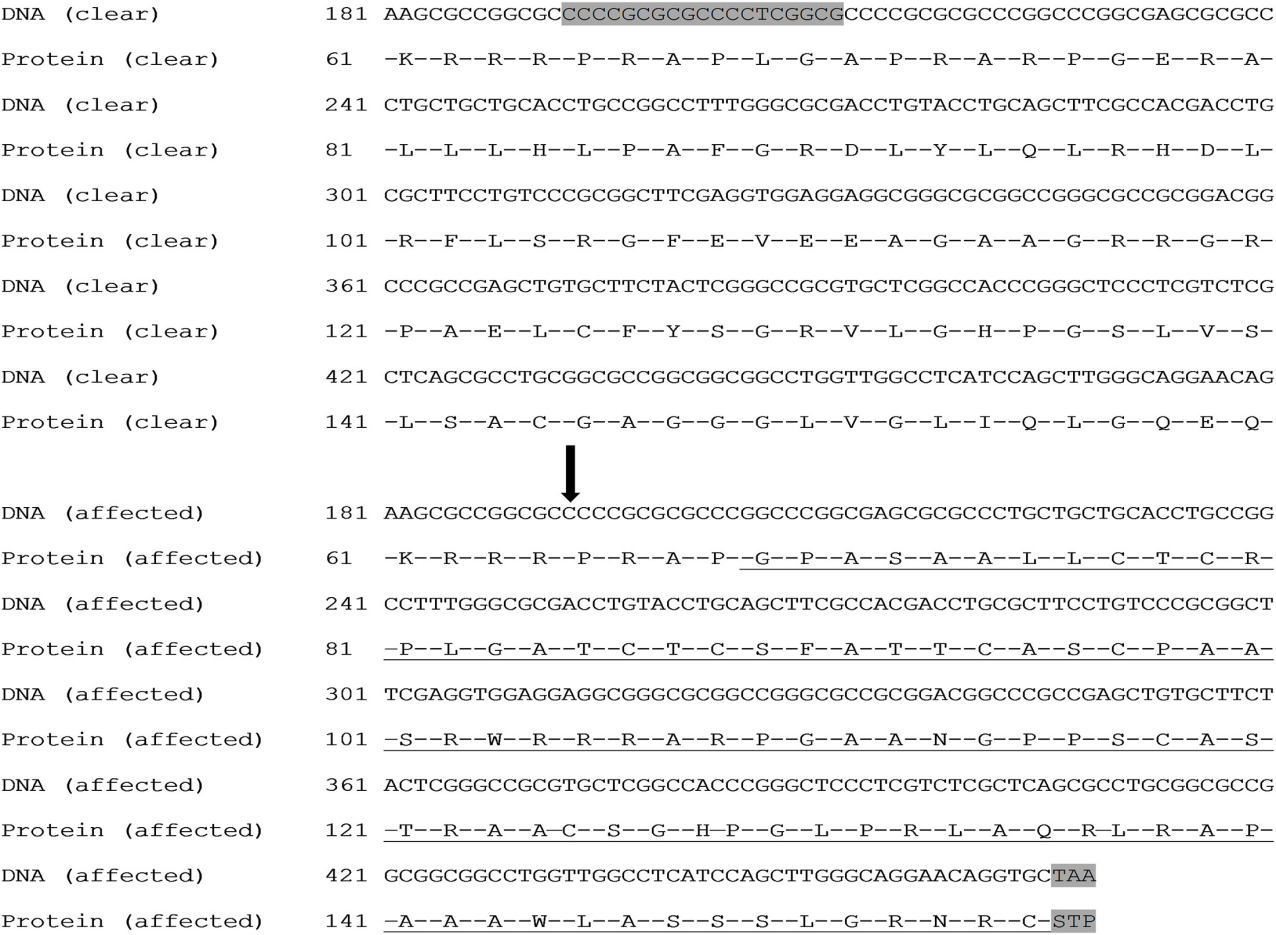
DNA and corresponding protein sequences in a normal Basset Hound (top) and one with POAG (bottom). The position of the deletion is indicated with a black arrow and the 19 deleted bases are indicated by shading in the DNA sequence of the clear dog. The deletion generates a frame shift leading to 87 aberrant amino acids (underlined) which introduces a premature stop codon, indicated by shading in the POAG-affected dog.

Visual scanning of the sequence read alignments from clinically affected Basset Hounds revealed only one variant that segregated with disease in the three affected dogs. This variant was a homozygous 19 bp deletion in exon 2 of *ADAMTS17* (CanFam3.1 chr3:40,614,853–40,614,872, Fig 1a) and was confirmed by Sanger sequencing (Fig 1b). The deletion is predicted to alter the reading frame of the gene, leading to 87 aberrant amino acids before introducing a premature stop codon at the 5’ end of exon 3, a predicted truncation of 86.1% of the protein (Fig 2). Fifty clinically unaffected Basset Hounds over the age of 72 months (controls) were genotyped for this allele; all were homozygous for the wild type. Genotyping of 173 additional Basset Hounds recruited for a different study and also known to be free of clinical signs of POAG, revealed 137 were homozygous for the wild type allele and 36 were heterozygous for the mutation. The frequency of the deletion in this subset of 223 Basset hounds was therefore 0.081 and the predicted frequency of affected dogs in the population estimated to be 0.007. One hundred and fifty two dogs of unrelated breeds (mixed breed panel) were all homozygous for the wild type allele. Following identification of this mutation, the owners of all affected dogs were contacted and pedigrees were obtained and reviewed. Analysis of these revealed two of the affected dogs to be full siblings although the third affected dog shared no common ancestors in its five generation pedigree. Taking into account the presence of the two full siblings, the association statistic between the deletion and POAG based on the entire genotyping dataset was p = 1.26 x 10^−10^.

#### Basset Fauve de Bretagne

**Fig 3:**
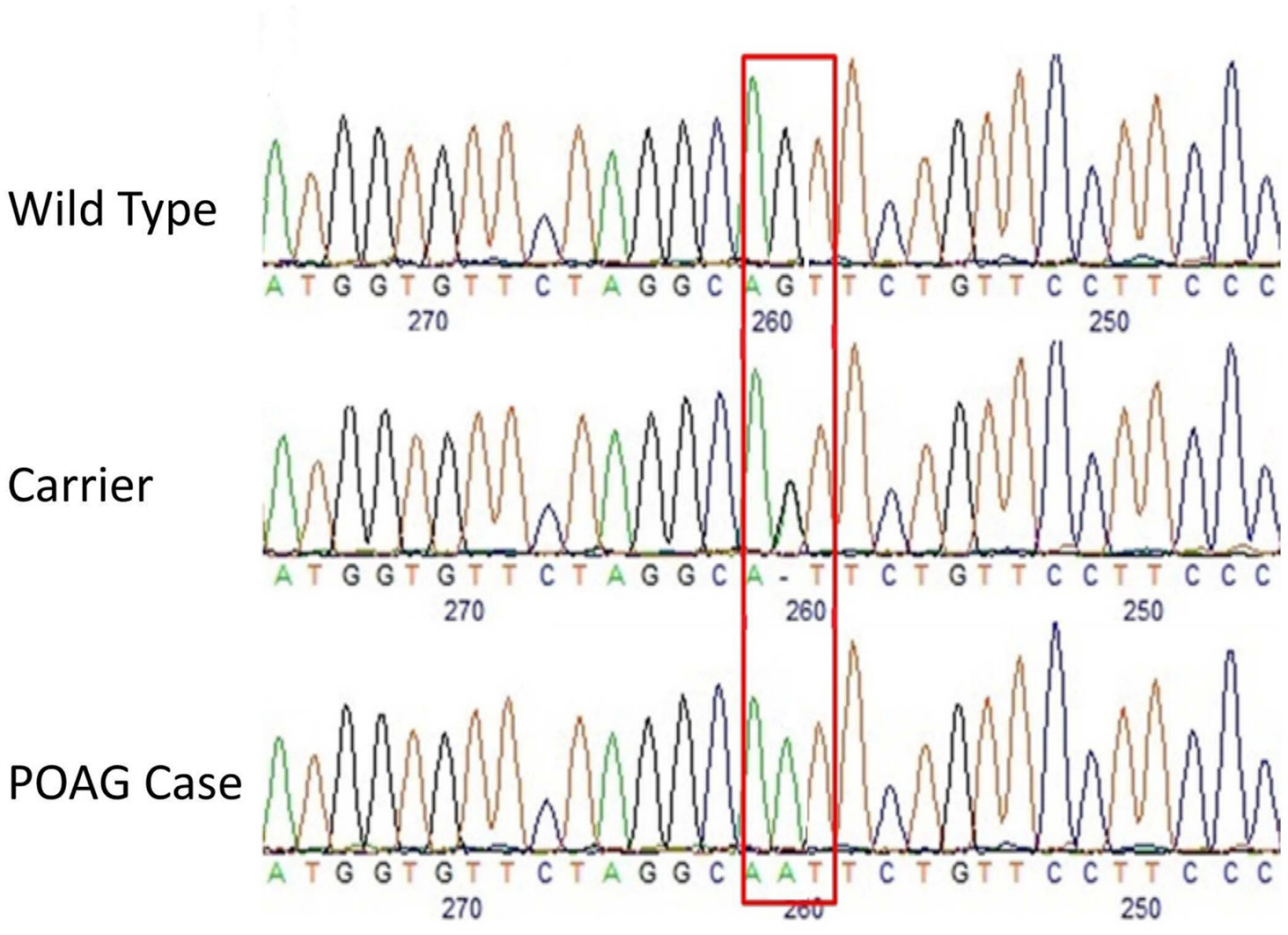
Electropherograms depicting the site of the missense mutation in the Basset Fauve de Bretagne affected with POAG. The red box depicts the exact site of the mutation (CanFam3.1 chr3:40,808,345). At this location, the wild type is GG, the carrier is AG and the POAG case is GG.

Visual scanning of the sequence read alignments from clinically affected Basset Fauve de Bretagne dogs revealed only one variant that segregated with disease—a homozygous G>A substitution in exon 11 of *ADAMTS17* (CanFam3.1 chr3:40,808,345, Fig 3). All affected Basset Fauve de Bretagne dogs were homozygous for this non-synonymous SNP which causes a glycine to serine (G519S) amino acid change in the disintegrin-like domain of ADAMTS17 and is predicted to be deleterious by SIFT [30]. Sequencing of 24 clinically unaffected Basset Fauve de Bretagne dogs revealed five were heterozygous for the mutation and 19 were homozygous for the wild type allele. The association statistic of the variant with POAG based on the entire genotyping dataset was p = 2.80 x 10^−7^. Genotyping of 85 dogs of unrelated breeds (mixed breed panel) and 90 dogs of related breeds (Basset Hound, Wire Haired Dachshund, Petit Basset Griffon Vendeen, Grande Basset Griffon Vendeen) for this variant with a TaqMan allelic assay revealed them all to be homozygous for the wild type allele.

## Discussion

Our study investigated *ADAMTS17* as a candidate gene for POAG in two different dog breeds—the Basset Hound and Basset Fauve de Bretagne. We considered *ADAMTS17* to be a strong candidate gene for study for several reasons. Firstly, a different mutation in *ADAMTS17* has recently been reported to cause POAG in another dog breed—the Petit Basset Griffon Vendeen [21]. Secondly, another *ADAMTS17* mutation is known to cause another canine ocular phenotype, primary lens luxation, which is characterised by structural abnormalities and weakness of the lens zonular fibres with ultimate dislocation of the lens from the patellar fossa (Table 1) [19,20]. The ciliary body is not only the site of origin of the lens zonular fibres and involved in their ongoing maintenance but it also contributes to IOP. The ciliary body is both responsible for aqueous humour production and also forms the ciliary cleft via which aqueous humour is drained from the eye into the systemic circulation. It is also already known that *ADAMTS17* is expressed within the ciliary body [19]. Thus, with consideration, it would not be surprising for different mutations within this gene to be associated with multiple intraocular phenotypes relating to abnormal function of the same anatomical region of the eye [19]. Thirdly, *ADAMTS17* mutations cause Weill-Marchesani and Weill-Marchesani-like syndromes in man [22,23]. These syndromes are rare connective tissue disorders which are characterised by various intraocular phenotypes including ectopia lentis, spherophakia, myopia and glaucoma along with joint stiffness and brachydactyly (Table 1). Clinical and genetic studies of these diseases suggest that *ADAMTS17* also plays a role in lens zonules and connective tissue formation in man [22,23]. Finally, mutations in closely related genes, *ADAMTS10* and *ADAMTSL4*, are known to be involved in primary glaucoma and other ocular connective tissue disorders in both man and dog [14–16,18,22,24,25].

**Fig 4:**
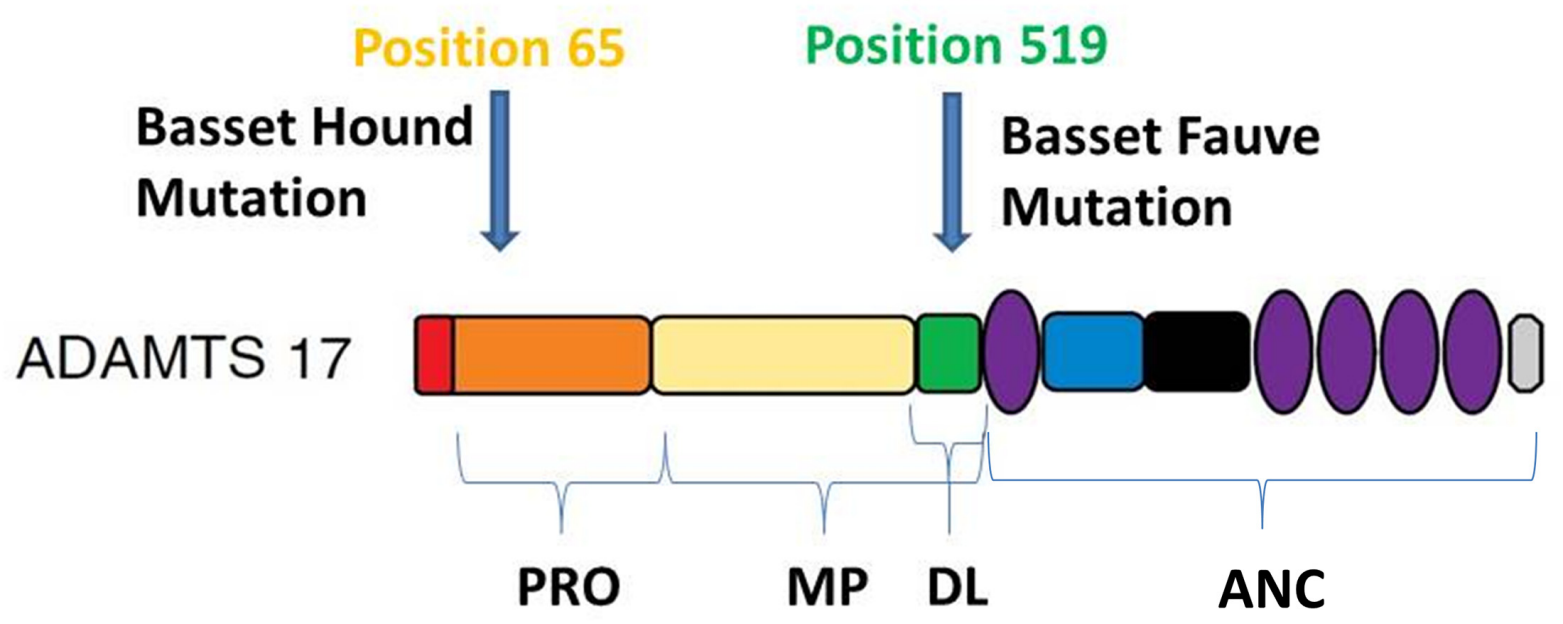
*ADAMTS17* protein structure denoting amino acid positions of the Basset Hound and Basset Fauve de Bretagne POAG mutations. ADAMTS17 is composed of a signal peptide (SP), prodomain (PRO), catalytic domain (CAT) (composed of the metalloproteinase (MP) and disintegrin-like domains (DL)) and an ancillary domain (ANC). The Basset Hound deletion corresponds to amino acid position 65 which is located in PRO. The Basset Fauve de Bretagne mutation corresponds to amino acid position 519 which is located in DL. Adapted from Kelwick et al. [31]

In the Basset Hound, a homozygous 19 bp deletion in exon 2 was present in all POAG-affected dogs. The deletion is predicted to alter the reading frame of the transcript and introduce a premature stop codon at the 5’ end of exon 3, which would result in a truncated and aberrant protein if the RNA is stable and not subjected to nonsense-mediated decay (Fig 2). If the transcript is translated the protein is expected to be truncated by 86.1% which would include the entire catalytic domain which is expected to lead to complete loss of protein function (Fig 4). Further supportive evidence for the deletion as the causative mutation of POAG in the Basset Hound was derived from genotyping. Our genotyping cohort included 223 Basset Hounds which had undergone complete ophthalmic examination and were found to be free from clinical signs of POAG at the time. 50 of these were significantly older than the POAG cases and were thought to be at very low risk of developing POAG in the future. These dogs served as controls and none were found to be homozygous for the identified mutation. Genotyping of the entire cohort allowed calculation of carrier frequency within the breed within the United Kingdom. These dogs were all resident and distributed widely around the United Kingdom and DNA had been collected from them for a parallel study. They can therefore be considered a random cohort with respect to the current study and representative of the Basset Hound population in the UK. The frequency of the mutant allele (0.081) and expected frequency of dogs affected with POAG (0.007) are fairly high. These data, together with the fact that POAG is a painful and blinding condition, underpin the importance of this mutation. With this in mind, a DNA test has been developed to allow Basset Hound breeders to genotype their dogs prior to breeding.

**Fig 5:**
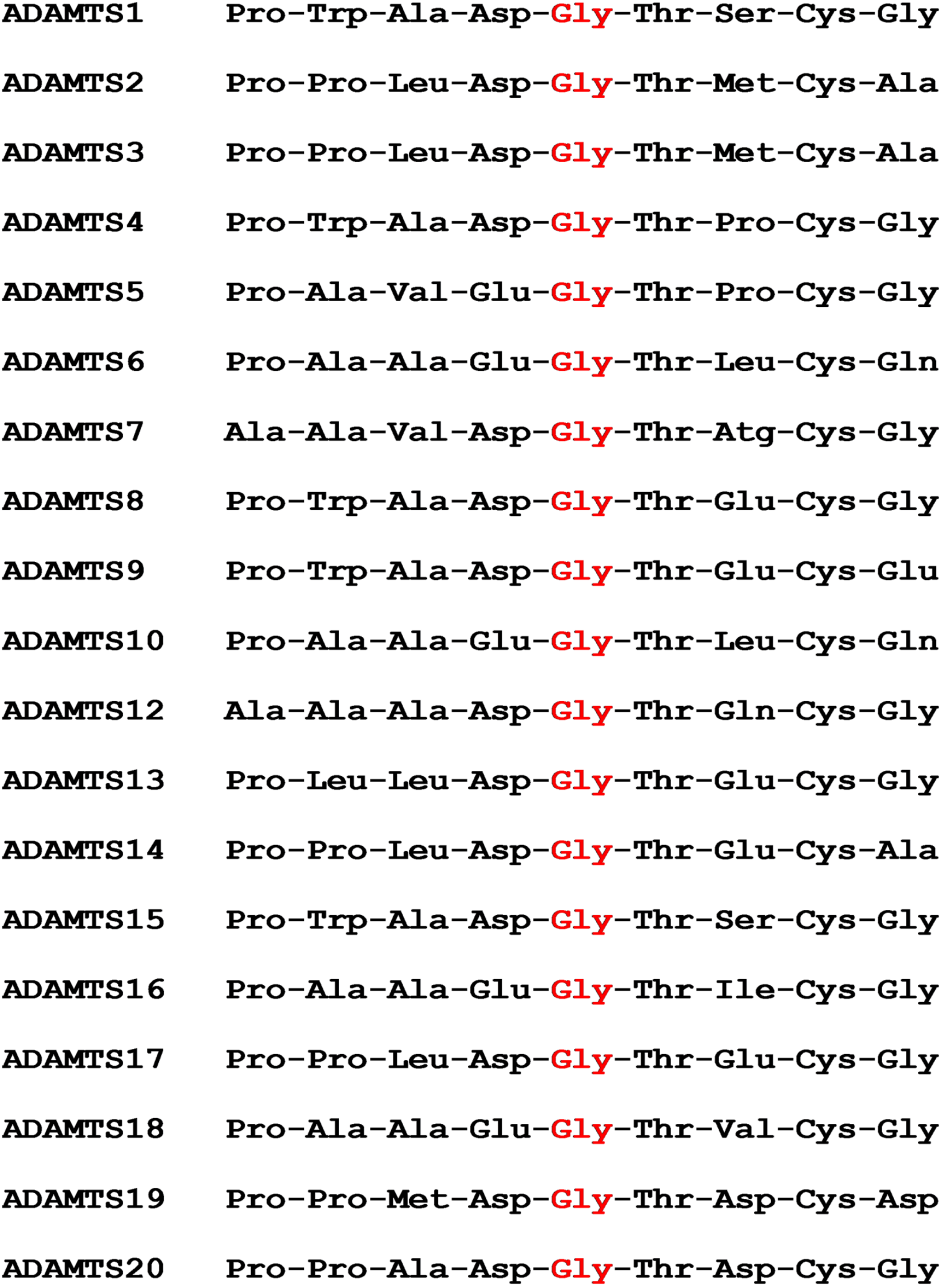
A selected region of the amino acid sequence of the disintegrin-like domain in the entire *ADAMTS* family. The region is centred on the glycine residue (in red) which is highly conserved and is the site of the missense mutation in the Basset Fauve de Bretagne.

**Fig 6:**
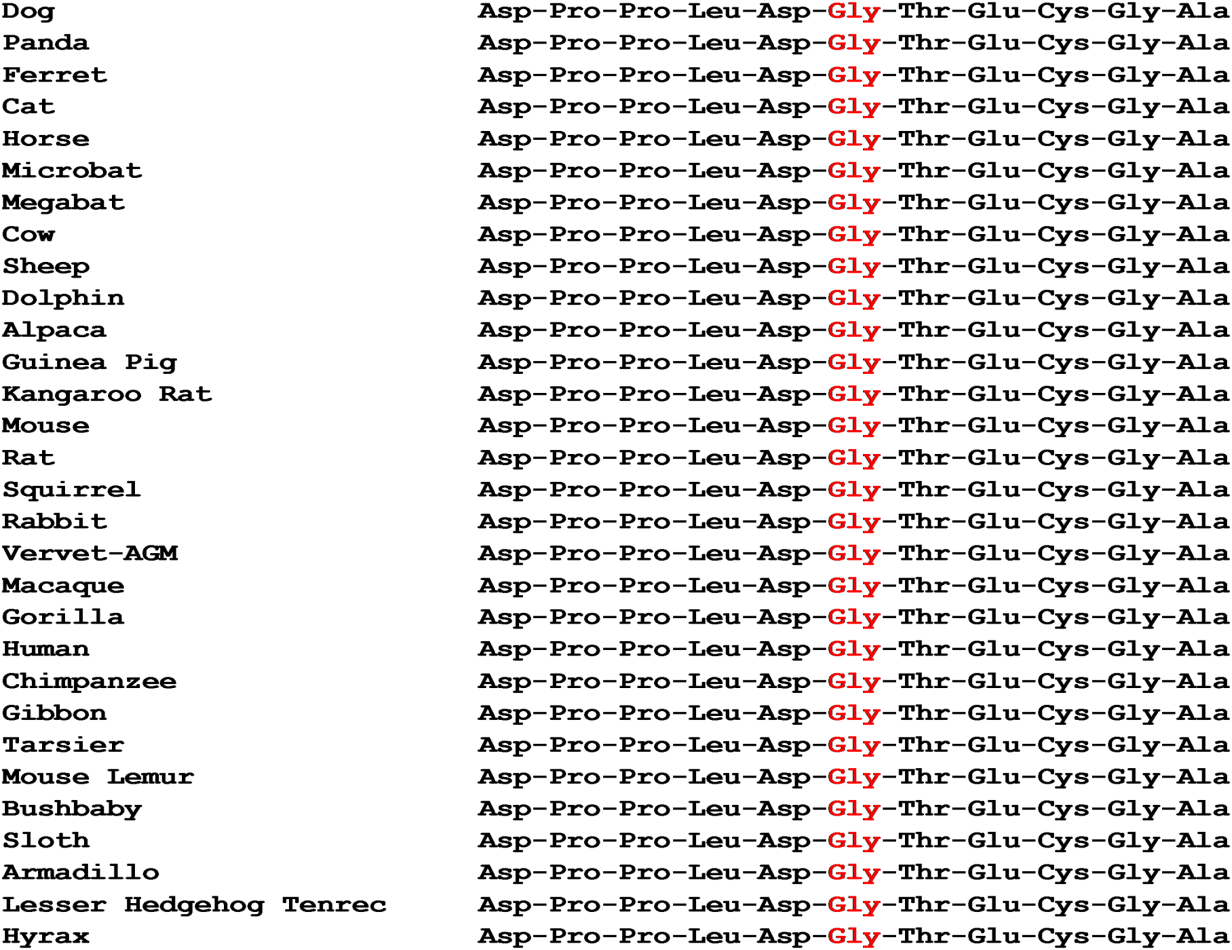
A selected region of the amino acid sequence of the disintegrin-like domain in *ADAMTS17* in 31 species. The region is centred on the glycine residue (in red) which is highly conserved and is the site of the missense mutation in the Basset Fauve de Bretagne.

The variant we have identified that is associated with POAG in the Basset Fauve de Bretagne is a non-synonymous SNP that results in a glycine to serine amino acid change in the disintegrin-like domain of the protein which is essential for the catalytic function of the protein [31] (Fig 4). Glycine is a highly conserved residue within the disintegrin-like domain across the entire ADAMTS family and also across species (Figs 5 and 6), providing strong evidence that this SNP is causal for POAG in this breed. Furthermore, 85 dogs of unrelated breeds and 90 dogs of related breeds were all homozygous for the wild type allele indicating that this variant is not a common polymorphism within the wider canine population.

ADAMTS17 (a disintegrin and metalloproteinase with thrombospondin type 1 motifs) is a member of a family of 19 known mammalian zinc-dependent metalloproteinases. Like other matrix metalloproteinases (MMPs), ADAMTSs have a role in extracellular matrix (ECM) degradation and turnover as well as in proteolysis of cell surface and soluble proteins [30, 31]. All ADAMTSs are secreted, extracellular enzymes that have a compound domain organisation comprising, from the amino terminus: a signal peptide followed by a pro-domain; a catalytic domain; and an ancillary domain that contains one or more thrombospondin type 1 repeats [31,32] (Fig 4). The pro-domain, amongst other functions, maintains latency and direct proper folding of the enzyme. The catalytic domain is comprised of metalloproteinase and disintegrin-like modules and confers the protease activity of the protein. The ancillary domain determines substrate specificity, localisation of the protease and its interaction partners [23,31,32]. ADAMTS-like genes (*ADAMTSL*) encode proteins that resemble the ancillary domains of ADAMTS but lack their catalytic domains and it is thought that these proteins may modulate the activities of the ADAMTSs [32,33].

The exact functions of ADAMTSs and ADAMTSLs are unknown. There is, however, strong support of a functional association between ADAMTS proteins and fibrillin microfibrils. Mutations in *ADAMTS10*, *ADAMTS17* and *ADAMTSL4* result in phenotypes that resemble those caused by mutations in fibrillin-1 (FBN1) [22,24,34–36]. It is thus suggested that ADAMTS and ADAMTSL proteins are involved either in microfibril assembly, stability and anchorage or the formation of function-specific supramolecular networks having microfibrils as their foundation [37]. How this functional relationship might be involved in POAG is unclear. The ECM of the TM is thought to be important in regulating intraocular pressure (IOP) [38]. In response to elevated IOP, specific proteases including MMPs are thought to be released by TM cells and are activated to degrade selected ECM molecules, leading to decreased resistance to aqueous humour outflow [39,40]. Furthermore, ADAMTS4 mRNA levels increase in response to increased IOP and recombinant ADAMTS4 increase outflow facility in human and porcine anterior segments [25]. Thus, loss of function mutations in genes which encode the proteins involved in ECM degradation are likely to be associated with elevated IOP and glaucoma. Further work, however, is required to determine if ADAMTS17 is indeed one of these proteins and to further understand POAG pathogenesis.

In conclusion, we report two separate mutations in *ADAMTS17* that are both strongly associated with the same ocular phenotype, POAG, in two different dog breeds. This report provides further evidence of the importance of *ADAMTS17* in intraocular physiology and increases the total number of reported canine *ADAMTS17* mutations to four (Table 1).

## Supporting Information

S1 Table PCR Primers for *ADAMTS17* Resequencing. (PDF)

S2 Table Primer pairs for amplification of candidate causal variants in *ADAMTS17*. (PDF)

S3 Table Allelic discrimination primers and probes. (PDF)
