# Correction: Two Independent Mutations in *ADAMTS17* Are Associated with Primary Open Angle Glaucoma in the Basset Hound and Basset Fauve de Bretagne Breeds of Dog

**DOI:** 10.1371/journal.pone.0156192

**Published:** 2016-05-18

**Authors:** James A. C. Oliver, Oliver P. Forman, Louise Pettitt, Cathryn S. Mellersh

Fig 3 is incorrect. The authors have provided a corrected version here.

**Fig 3:**
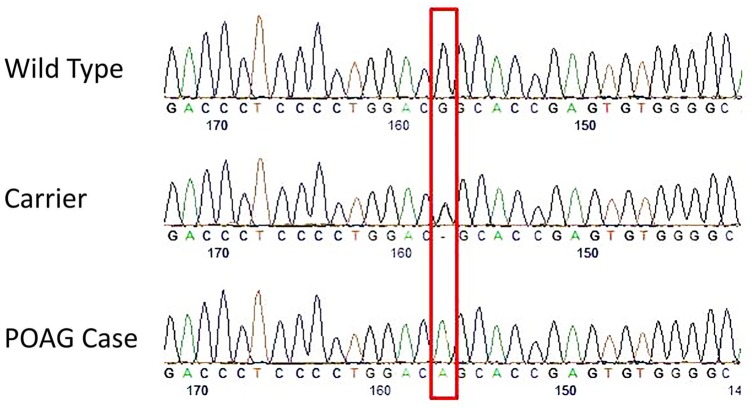
Electropherograms depicting the site of the missense mutation in the Basset Fauve de Bretagne affected with POAG. The red box depicts the exact site of the mutation (CanFam3.1 chr3:40,808,345). At this location, the wild type is GG, the carrier is AG and the POAG case is AA.
