# Supplementary material for: Two Independent Mutations in ADAMTS17 Are Associated with Primary Open Angle Glaucoma in the Basset Hound and Basset Fauve de Bretagne Breeds of Dog
Source: PLoS One. 2015 Oct 16;10(10):e0140436. doi: 10.1371/journal.pone.0140436 (PMC4608710; doi:10.1371/journal.pone.0140436)
Supplement: S1 Table — (PDF) [file pone.0140436.s001.pdf]

Target	Forward Primer Sequence/Reverse Primer Sequence	Amplicon Size (bp)	Annealing T (°C)
Exon 1	TGATTTACCACGTTGGGTTTG/GGGAGGGCAGGAGGAAGGT	273	56
Exon 2 (5′)	GCTGACGCGTCTCCTCTCTCCC/CCGCCTCCTCCACCTCGAA	285	56
Exon 2 (3′)	CCCCGGACCCCGAAAGC/GCGACTAAGCGACGGGCAGA	334	56
Exon 3	ACATGAGGACCAGGCCAGA/AGGGCTGCTACACATGAAATG	474	56
Exon 4	TTCGATGTGCCTCAGCTCTAC/GACCCAGGCACTGAAACTACA	591	56
Exon 5	CCAACATCTTCCTCTGTTCCA/GGAGAGCAGACAAGACTGACAA	300	56
Exon 6	CATGACCTGATCAACCACTGA/TTACTGATGAGGATGCCAAGG	558	56
Exon 7	ATTGCTATGTGCAGGATGACC/GCAACAGGAAAGGCAGAGTTT	293	56
Exon 8	GGTGAATCCCAAAGCATTACA/GTAATCTCTCCCGTTCCCTGA	488	56
Exon 9	GTAGCCAAGTACAGGGCATCA/CCTGGGAGAAATGAAGTAGGG	567	56
Exon 10	TCCAATGCCTGAGTCATCTTC/AACTGCCTGTGAGGGTGTATG	434	56
Exon 11	TGAATTCCAAGTCCAAACCAG/CCAGTGGAGCTTTAGGCACTAT	315	56
Exon 12	TGCAGTGATCTGGTGAGTGAG/GCTTTGTTGAAGCTGAGATGC	426	56
Exon 13	GGAGGTTGCTTTGGAAACTCT/ACTCTCCCAGAGTTGGGTCAT	490	56
Exon 14	GAACATGTGCTGGGTTTCTGT/GTGGGCTTTATGCTCAGTCAC	490	56
Exon 15	CCTAGGCACCAACACTTGCT/GCCTTTCAGCAAGCATAACAC	450	58
Exon 16	CTCAGAGTCAGACCAGCCTGT/GCTTCCTTCCCTCTGTCATCT	468	56
Exon 17	CTGTGAGCCAGTCTTCCATTC/ACCAGAACCCAGGTGATCTCT	432	56
Exon 18	TCTGAGGAACCCAAGAGTGAA/GTTCCTGTGGAGAGACAGGTG	449	56
Exon 19	GCCATGTCTTACACACCCTCA/GCAAGCAGAAGTCACTTAGCAA	468	56
Exon 20	TGAGTACATTTCCCTCCCTCA/GGCAAGGACTGTGATACTTGG	458	56
Exon 21	TCAGACTCTAGATGCCCAGGA/CTCTAGGGAGCATTGGGTTTC	490	56
Exon 22	AGCCTCCTTGTCCTGCATTAT/AATCCCATCTCTGCAACCTCT	489	56
Exon 23	CGAGTGAGGGCAGCTTAGAGT/TCAGGTTCACGCTCAAGTTCT	469	56
