# Supplementary material for: Two Independent Mutations in ADAMTS17 Are Associated with Primary Open Angle Glaucoma in the Basset Hound and Basset Fauve de Bretagne Breeds of Dog
Source: PLoS One. 2015 Oct 16;10(10):e0140436. doi: 10.1371/journal.pone.0140436 (PMC4608710; doi:10.1371/journal.pone.0140436)
Supplement: S2 Table — (PDF) [file pone.0140436.s002.pdf]

Target	Forward Primer Sequence/Reverse Primer Sequence	Amplicon Size (bp)	Annealing T (^oC)
Exon 2 19 bp deletion	ACGTGGAGGTGGTGGTGCTG/CGAAGCTGCAGGTACAGGTC	190	60
Exon 11 SNP	ATCCAGATTCACAACCCTCCT/GGAGATAGAAGTGTTCCTCTTTGC	409	57
