# Supplementary material for: Two Independent Mutations in ADAMTS17 Are Associated with Primary Open Angle Glaucoma in the Basset Hound and Basset Fauve de Bretagne Breeds of Dog
Source: PLoS One. 2015 Oct 16;10(10):e0140436. doi: 10.1371/journal.pone.0140436 (PMC4608710; doi:10.1371/journal.pone.0140436)
Supplement: S3 Table — (PDF) [file pone.0140436.s003.pdf]

Assay name	Forward Primer Sequence	Reverse Primer Sequence	Reporter 1 Sequnce (5'VIC)	Reporter 2 Sequence (5'6-FAM)
BFDBPOAG	CTGCAAGACCAAGCTGGA	ACTATTGAGTACAGTGCAGACCAC	CCTGGACGGCACCGAGT	CCCTGGACAGCACCGAGT
